# Iron deficiency and iron deficiency anemia in inherited bleeding disorders: common, underrecognized, and undertreated

**DOI:** 10.1016/j.rpth.2025.103187

**Published:** 2025-09-18

**Authors:** Hanny Al-Samkari

**Affiliations:** 1 Division of Classical Hematology, Mass General Brigham Cancer Institute, Massachusetts General Hospital, Boston, Massachusetts, USA; 2 Harvard Medical School, Boston, Massachusetts, USA

Recently in *Research and Practice in Thrombosis and Haemostasis*, Dreier et al. [1] present a study describing the prevalence of iron deficiency (ID) in patients with mild-to-moderate inherited bleeding disorders (including von Willebrand disease, inherited platelet disorders, and rare coagulation factor deficiencies, but not including patients with mild-to-moderate vascular bleeding disorders, such as mild-to-moderate hereditary hemorrhagic telangiectasia) and bleeding disorders of unknown cause. In their study, the prevalence of ID and iron deficiency anemia (IDA) was assessed in the Vienna Bleeding Biobank and compared with age-and-sex-matched healthy controls. ID was defined conservatively, as a serum ferritin of <30 ng/mL and/or transferrin saturation of <16%. They found a prevalence of ID of 39% in patients with any bleeding disorder and 49% in the subgroup of patients with von Willebrand disease, compared with 31% in age-and-sex-matched controls. The prevalence of IDA was similar in both groups. Both female sex and increased body mass index were patient characteristics significantly associated with a higher odds of having ID in the analyzed bleeding disorder population.

While this is not the first study to examine this topic [[2], [3], [4], [5]] and other studies have found prevalence rates of ID and IDA in excess of 50% in various inherited bleeding disorder populations, it should come as something of a surprise to the hemostasis and thrombosis scientific community that this is not a topic that has been thoroughly investigated, even in 2025. Therefore, the efforts of Dreier et al. [1] and their study are a welcome addition to the literature on such a common, readily diagnosed, and easily managed issue that degrades the quality of life of so many. Forgive me if you, as a reader, do not need to be reminded of the many negative consequences of chronic ID, but I will do so, nevertheless, because there is an ongoing worldwide epidemic of missed (or worse, neglected) ID, particularly in women. The epidemic of ID is one of the leading causes of missed days of productivity and years lived with disability worldwide [6]. It results in the symptoms not only we all think of commonly—reduced energy, fatigue, and impaired exercise tolerance—but also those that many of us forget: poor sleep, restless legs syndrome, hair loss, mind fog resulting in temporary cognitive impairment until iron is repleted (enough to temporarily reduce intelligence quotient) [7,8], and increased thromboembolic risk [[9], [10], [11]]. Moreover, the latter, of course, is particularly relevant for those with bleeding disorders, for whom treatment of thromboembolism of any kind is much more complicated. The most significant limitation of the study by Dreier et al. [1] is the lack of data pertaining to any prior oral or intravenous iron supplementation, and its timing, prior to study enrollment. The authors acknowledge that the lack of this knowledge, along with other limitations in the study (such as interventions to reduce bleeding prior to study enrollment), may have led to an underestimation of ID and IDA in these patients. I would take it a step further and conclude that such an underestimation is a near certainty given these limitations and the overly conservative definitions used for ID in this study (serum ferritin of <50 ng/dL or transferrin saturation of <20% are more appropriate thresholds in the light of modern evidence and would be quite a bit more sensitive [12,13]). The fact remains that ID and IDA remain underrecognized and undertreated in the general population (to the detriment of over a billion people worldwide), especially in women. In patients with bleeding disorders, this is an even greater problem (again, especially in women).

It is the responsibility of all of us in the professional medical community to do our part in rectifying this problem. Among those of us in the hematology and hemostasis–thrombosis medical community, we must set the standard for others to follow. Screening for ID and IDA is inexpensive, easy, and, given the results of the work by Dreier et al. [1] as well as other studies, should be considered mandatory in people with inherited bleeding disorders as well as bleeding disorders of unknown cause. The potential to provide benefit for patients is high, and the risk approaches zero. For women of childbearing potential, prompt diagnosis and management of ID and IDA is all the more crucial, given the constant burden of menstrual blood loss and the recognized importance of maternal iron sufficiency on neurologic development of the fetus [13]. Maternal ID is associated with the development of autism, schizophrenia, and abnormal brain structure in the offspring [14], invoking the specter of a lifetime of mental disease being potentially preventable by 1 iron infusion prior to pregnancy. While serum ferritin alone is an imperfect measure of iron sufficiency, for individuals with bleeding disorders who have no acute or chronic inflammation, aiming for a serum ferritin goal of greater than 50 ng/mL is prudent, and this threshold is highlighted in various clinical practice guidelines [13,15]. This is particularly true if patients have chronic ongoing bleeding, as is often the case in hereditary hemorrhagic telangiectasia and von Willebrand disease [16], and in any menstruating woman irrespective of the presence of an underlying bleeding disorder.

Treatment of ID and IDA in persons with bleeding disorders should be prompt and more aggressive than in the general population given the ongoing blood loss and/or potential future blood loss (Figure). While most ID guidelines recommend beginning with oral iron in most patients (except those with more severe anemia, in whom going directly to intravenous iron is recommended), clinical judgment must guide this decision, and starting with intravenous iron is prudent in many, if not most, patients with chronic bleeding [16]. Most patients (∼70%) develop unpleasant side effects when taking oral iron, including constipation, dyspepsia, nausea, and blackened or dark stools [17,18], drastically impacting compliance and/or worsening patient quality of life. The amount of iron absorbed from each oral iron pill is a tiny fraction of the iron present in the pill, generally not more than a few milligrams; this amount is less in older patients and those using any antacid medications due to hypochlorhydria. It is common for patients to begin over-the-counter antacid medication to combat the gastrointestinal upset caused by the oral iron itself, unknowingly self-sabotaging the absorption of the oral iron. Many patients with mild or moderate bleeding disorders “tread water” on oral iron—that is, their serum ferritins range between 15 and 40 ng/mL but they are never, or not consistently, actually replete. This is not acceptable and merits administration of intravenous iron (which is often required on an intermittent basis to maintain proper iron sufficiency). While oral iron may be suitable for patients with bleeding disorders and mild ID or IDA and who tolerate it and absorb it well, patients with IDA who fail to achieve an increase in hemoglobin of at least 1 to 2 g/dL after 4 to 6 weeks (with hemoglobin normalization by 12 weeks) and those with ID who fail to consistently maintain a ferritin >50 ng/dL and transferrin saturation of >20% should be promptly switched to intravenous iron and repleted with at least 1000 mg intravenous elemental iron as initial infusional therapy. Patients with bleeding disorders who continue to chronically bleed universally require intermittent retreatment to avoid plunging back into ID and IDA.

**Figure:**
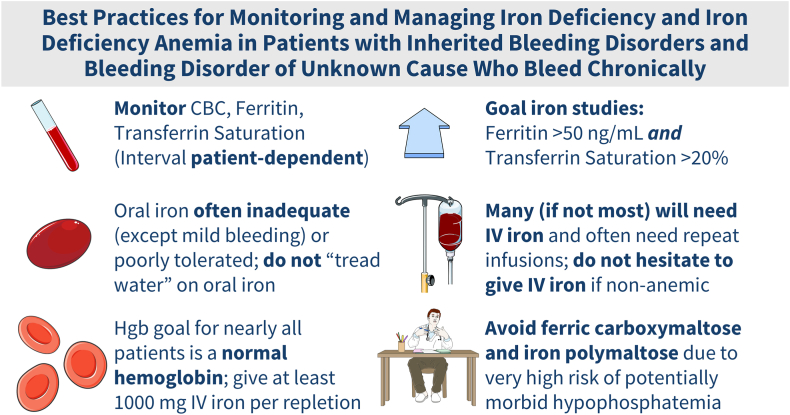
Best practices in the management of iron deficiency and iron deficiency anemia in patients with inherited bleeding disorders and chronic bleeding. CBC, complete blood count; IV, intravenous.

Any intravenous iron formulation—new or old, expensive or inexpensive—is just fine to use, so long as adequate quantities of elemental iron are given, with the important caveat that ferric carboxymaltose and iron polymaltose are avoided. Ferric carboxymaltose in particular carries an unacceptably high risk of treatment-emergent hypophosphatemia (50%-75% with a single treatment course), which blunts the benefits of iron repletion from a symptomatic standpoint and can lead to severe complications (including hypophosphatemic osteomalacia and skeletal insufficiency fractures) in those patients requiring repeat infusions over time [19], which certainly includes patients with bleeding disorders. But, for all other formulations currently on the market—particularly low-molecular-weight iron dextran, iron sucrose, ferumoxytol, and ferric derisomaltose—there is virtually no risk of significant hypophosphatemia, the risk of anaphylaxis is vanishingly small (contrary to what is taught in medical school and residency), and these formulations are extremely safe [18]. But, perhaps, the most important issue to address regarding iron therapy in patients with bleeding disorders is that it is almost impossible to “overtreat” with intravenous iron unless one is constantly administering numerous unnecessary infusions (totaling several thousand milligrams of elemental iron) in a patient who is not actively bleeding and has elevated ferritin values, which of course is not done. The real risk, once again, is undertreatment, as the study by Dreier et al. [1] demonstrates. Hopefully, this study will further raise awareness of the under recognition and undertreatment of ID and IDA in our patients with bleeding disorders and improve the quality of life and health outcomes of these patients.
